# Detection of persistent organic pollutants binding modes with androgen receptor ligand binding domain by docking and molecular dynamics

**DOI:** 10.1186/1472-6807-13-16

**Published:** 2013-09-22

**Authors:** Xian Jin Xu, Ji Guo Su, Anna Rita Bizzarri, Salvatore Cannistraro, Ming Liu, Yi Zeng, Wei Zu Chen, Cun Xin Wang

**Affiliations:** 1 College of Life Science and Bioengineering, Beijing University of Technology, Beijing 100124, China; 2 College of Science, Yanshan University, Qinhuangdao 066004, China; 3 Biophysics and Nanoscience Centre, Facolta di Scienze, Universita della Tuscia, Largo dell’Universita, 01100 Viterbo, Italy; 4 Beijing Institute of Biotechnology, Beijing 100071, China

**Keywords:** Persistent organic pollutants, Androgen receptor, Molecular docking, Molecular dynamics, MM/PBSA

## Abstract

**Background:**

Persistent organic pollutants (POPs) are persistent in the environment after release from industrial compounds, combustion productions or pesticides. The exposure of POPs has been related to various reproductive disturbances, such as reduced semen quality, testicular cancer, and imbalanced sex ratio. Among POPs, dichlorodiphenyldichloroethylene (4,4’-DDE) and polychlorinated biphenyls (PCBs) are the most widespread and well-studied compounds. Recent studies have revealed that 4,4’-DDE is an antagonist of androgen receptor (AR). However, the mechanism of the inhibition remains elusive. CB-153 is the most common congener of PCBs, while the action of CB-153 on AR is still under debate.

**Results:**

Molecular docking and molecular dynamics (MD) approaches have been employed to study binding modes and inhibition mechanism of 4,4’-DDE and CB-153 against AR ligand binding domain (LBD). Several potential binding sites have been detected and analyzed. One possible binding site is the same binding site of AR natural ligand androgen 5α-dihydrotestosterone (DHT). Another one is on the ligand-dependent transcriptional activation function (AF2) region, which is crucial for the co-activators recruitment. Besides, a novel possible binding site was observed for POPs with low binding free energy with the receptor. Detailed interactions between ligands and the receptor have been represented. The disrupting mechanism of POPs against AR has also been discussed.

**Conclusions:**

POPs disrupt the function of AR through binding to three possible biding sites on AR/LBD. One of them shares the same binding site of natural ligand of AR. Another one is on AF2 region. The third one is in a cleft near N-terminal of the receptor. Significantly, values of binding free energy of POPs with AR/LBD are comparable to that of natural ligand androgen DHT.

## Background

**Figure 1:**
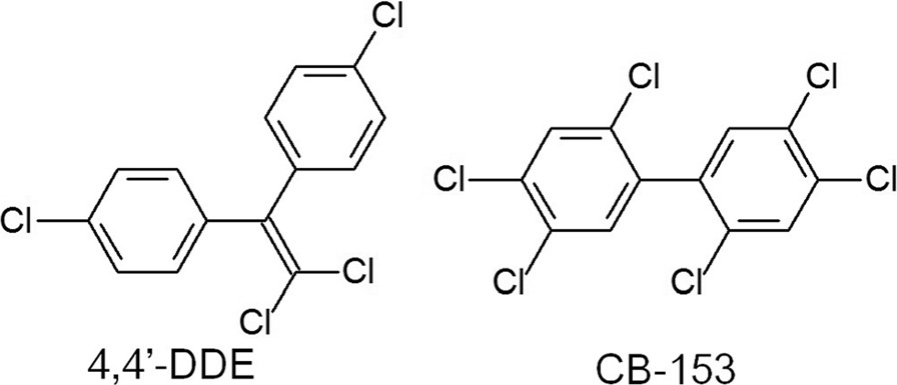
Structures of 4,4’-DDE and CB-153.

Persistent organic pollutants (POPs), mainly from industrial compounds, combustion productions or pesticides, widely exist in the environment and are considered to be potential endocrine disrupting chemicals (EDCs) [1-3]. These compounds are persistent in the environment after releasing and transported to human body mainly through contaminated foods [4]. Recent studies have linked POPs exposures to reproductive disturbances, such as reduced semen quality, testicular cancer, and imbalanced sex ratio [5-9]. Among POPs, dichlorodiphenyldichloroethylene (4,4’-DDE) and polychlorinated biphenyls (PCBs) are the most widespread and well-studied compounds. 4,4’-DDE is the major metabolite of the widely used organochlorine insecticide dichlorodiphenyltrichloroethane (DDT) and has been considered a good indicator of DDT exposure [10,11]. Among the 209 possible congeners of PCBs, 2,2’,4,4’,5,5’-hexachlorobiphenyl (CB-153) is the most common one and considered as a useful marker of PCBs [11,12]. The structures of 4,4’-DDE and CB-153 are illustrated in Figure 1. These compounds display substantial structural similarities with natural ligands of nuclear receptors (NRs) and thus are capable of disrupting their signaling pathways via direct binding to the receptor [13,14].

**Figure 2:**
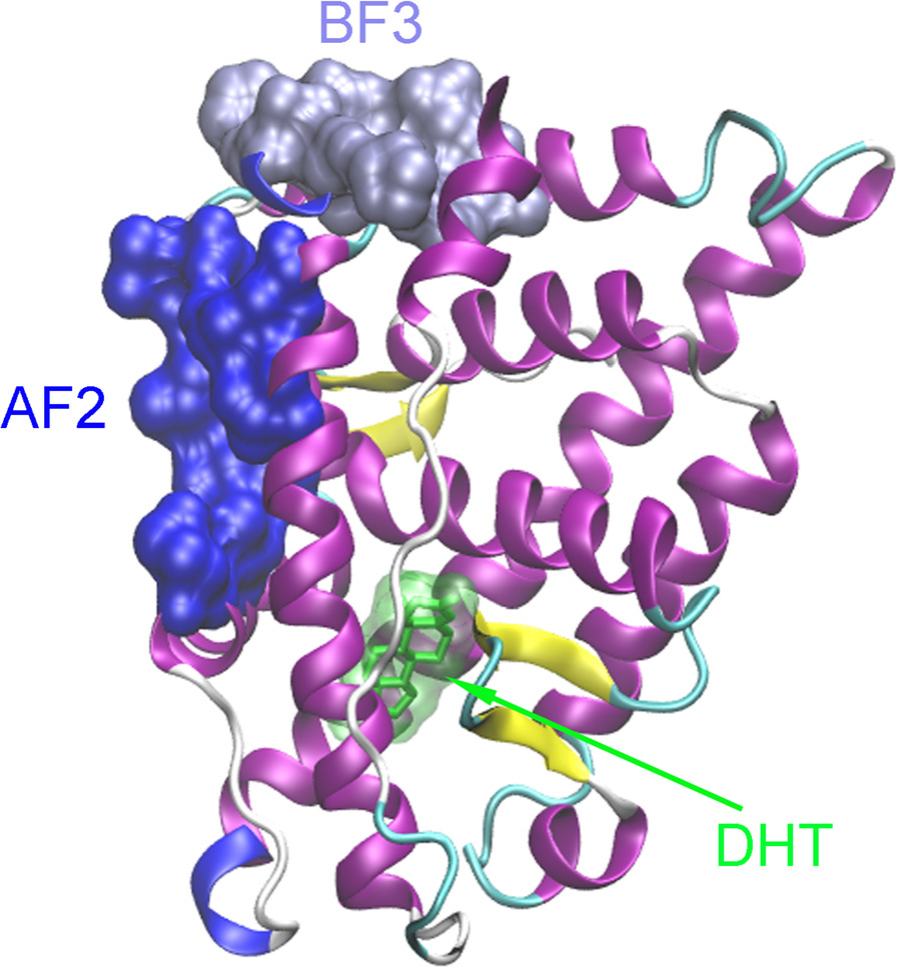
**Crystallographic structure of AR/LBD-DHT complex (PDB ID: 1I37).** The ligand DHT is represented with the licorice model and the receptor LBD is represented with the newcartoon model. AF2 and BF3 sites on LBD are displayed by the solvent accessible surface area model.

Androgen receptor (AR) is a ligand activated transcription factor belonging to nuclear receptor superfamily, which is involved in regulation of various physiological functions in human body, including cell growth, proliferation, and differentiation [15]. AR is a soluble protein and AR-regulated gene expression is responsible for the male reproductive system [16]. Its activity is regulated by the binding of either androgens testosterone or 5α-dihydrotestosterone (DHT). Like other members of this family, AR consists of three functional domains, the N-terminal transactivation domain, the central DNA binding domain (DBD), and the C-terminal ligand binding domain (LBD) that harbors a ligand-dependent transcriptional activation function (AF2) [17]. AF2 region is a hydrophobic cleft formed by the binding of agonist and then binds with steroid receptor coactivator (SRC) family of coactivators [16]. Since AR is considered as an effective therapeutic target of prostate cancer, various compounds have been designed as its inhibitors through directly binding to the ligand binding site avoiding natural ligands binding [18]. A recent study also revealed that several small molecules can bind to AF2 and then prevent the transcriptional activation of AR [19]. Studies on other members of the NR superfamily, such as estrogen receptors (ER) and progesterone receptor (PR), also revealed that the AF2 region on LBD is the binding site of some antagonists [20-23]. Besides, another novel binding site, binding function 3 (BF3), on the LBD have been identified by a recent virtual screening study combined with biochemical assays and X-ray crystallography [24,25] (see Figure 2).

Indeed, 4,4’-DDE has been found to be an antagonist of AR by numerous studies [14,26,27]. However, the mechanism of the inhibition remains elusive. With respect to CB-153, partial antagonistic properties on AR were detected by Schrader *et al*. [28], whereas Bonefeld-Jørgensen *et al*. found that CB-153 has no effect on AR [29]. In the present work, we employed computational approaches, molecular docking, molecular dynamics (MD), and binding free energy calculation, to explore detailed interactions between POPs and AR/LBD. The binding modes of 4,4’-DDE and CB-153 with AR/LBD were identified with molecular docking method, followed by long time MD simulations. The binding free energies of AR/LBD-POPs complexes were also calculated and compared with that of the natural ligand DHT. Based on these results, the inhibitory mechanism of POPs against AR was proposed.

## Methods

### Molecular docking

POPs were docked to AR/LBD based on crystal structure taken from Protein Data Bank (PDB) with PDB entry 1I37 [17]. Autodocktools was used to prepare the systems and the Gasteiger partial charges were assigned to the receptor and ligands [30]. Dockings were performed with AutoDock4.2 package [31]. Firstly, a box of 126 × 126 × 126 points was set with grid spacing 0.6 Å in each direction to make sure there is enough space to fit the whole receptor and also for the free rotation of ligands. The maximum number of energy evaluations was set to 2.5 × 10^7^. The Lamarckian genetic algorithm (LGA) was used for the sampling of complex conformations formed by ligand and receptor. Other parameters were set to default. Then, dockings were focused on predicted potential binding sites with a grid number of the box 60 × 60 × 60 and smaller grid spacing 0.375 Å. In docking calculations, single bonds of ligands were treated as rotatable. Two and one flexible torsions were defined for 4,4’-DDE and CB-153, respectively. A hundred receptor-ligand complexes were generated for each docking. Docking poses were further refined by long time MD simulations.

### Molecular dynamics simulations

MD simulations of AR/LBD and AR/LBD-ligand complexes were carried out by GROMACS4.5.5 [32] with GROMOS 43a1 force field [33]. Topologies of small molecules were generated by using PRODRG server [34]. Ligands were optimized using Gaussian 03 with Hartree-Fock/6-31G* level and then mulliken charges were reassigned to ligands for MD simulation [35,36]. Complexes were solvated by simple point charge (SPC) water [37] in a cubic box extending at least 1.0 nm in all directions from the solute. Five Cl^-^ ions were added to neutralize charges of the system. Long-range electrostatic interactions were calculated by the particle mesh Evald (PME) method [38]. A cutoff radius of 1.0 nm for van der Waals and short-range electrostatic interactions was used. LINCS algorithm was used to constrain bond lengths [39]. The temperature of the system was coupled by using the velocity rescaling method [40] and the pressure was coupled by using the Parrinello-Rahman method [41]. The integration time step was set to be 2 fs. First, the system was minimized for 1000 steps using the steepest descent algorithm, followed by a 200 ps position restrained molecular dynamics simulation. Then the system was heated from 50 K up to 300 K by steps of 50 K within 500 ps. Finally, a 30 ns simulation was carried out for each system at a constant temperature of 300 K and a constant pressure of 1 atm. Trajectories were analyzed by using GROMACS software package and the figures of the protein structures were created by VMD program [42].

### MM/PBSA binding free energy

MM/PBSA (Molecular Mechanics/Poisson-Boltzmann Surface Area) method [43], which combines molecular mechanics energy and continuum solvent models, has been widely used for the free energy calculation of receptor-ligand complexes [44,45]. The binding free energy, ΔG_b_, of a ligand (L) binding to a receptor (R) forming a complex RL is calculated as

(1) $$\Delta G_{b}=\Delta E_{\mathrm{MM}}+\Delta G_{\mathrm{solv}}-\mathrm{T\Delta S}$$

where the first term, ΔE_MM_, gives the gas phase molecular mechanics energy changes, which includes contributions from the internal (ΔE_int_), electrostatic (ΔE_elec_), and van der Waals (ΔE_vdW_) energies. The second term, ΔG_solv_, provides the changes of the solvation contributions consisting of the polar solvation energy (ΔG_PB_,) and nonpolar solvation energy (ΔG_SA_). In the present work, the ΔG_PB_ was calculated by Adaptive Poisson-Boltzmann Solver (APBS) program [46]. The grid spacing was set to 0.6 Å. The value of the exterior dielectric constant was set to 80, and the solute interior dielectric constant was set to 4 [47]. The atomic radii and charges were set according to those used in MD simulations. The ΔG_SA_ was determined by the solvent-accessible surface area (SASA) approach, *ΔG*_*SA*_ = *γ* × *ΔSASA* + *β*, with γ = 2.2 kJ/(mol nm^2^) and β = 3.84 kJ/mol [43,48]. The quasi-harmonic analysis was performed to estimate the entropy change (−TΔS) of the system upon ligand binding [49]. In the method, the entropy is calculated based on the all atom covariance matrix, which can be obtained using a standard GROMACS utility applied to a MD trajectory. The conformational entropy was estimated using the following expression

(2) $$S_{\mathrm{ho}}=k_{b}\sum_{i=1}^{3N-6}\left[\frac{\gamma }{e^{\gamma }-1}-\text{ln}\left(1-e^{\gamma }\right)\right]$$

where $\gamma =h/2\pi \sqrt{1/k_{B}T\lambda_{i}}$, with *h* is the Planck constant, *k*_*B*_ is the Boltzmann constant, T is the absolute temperature, and λ_i_ are eigenvalues of the all-atom mass-weighted covariance matrix of fluctuations $\delta_{\mathrm{ij}}=\sqrt{m_{i}m_{j}}\langle \left(x_{i}-\langle x_{i}\rangle \right)\left(x_{j}-\langle x_{j}\rangle \right)\rangle$.

In this work, 1000 snapshots extracted from the last 10 ns simulations were used in the binding free energy calculations of AR/LBD-POPs complexes.

## Results and discussions

### Docking of POPs to AR/LBD

DHT was redocked to AR/LBD with 100 independent runs and docking results were clustered according to the root mean square difference (RMS) with a cutoff value 0.2 nm. A large cluster (occupies 59% of total generations) with the lowest value of binding energy (−45.6 kJ/mol) was observed in which ligand binds to the natural ligand binding site. In the second largest cluster (24%), DHT binds to a region near the C-terminal of the receptor (PBS3 in the following text) with a much larger value of binding energy −30.4 kJ/mol. In remaining generations, DHT arbitrarily binds on the surface of AR/LBD with binding energies higher than −28.1 kJ/mol. Significantly, the redocking of DHT to the natural ligand binding site shows that the root mean square deviations (RMSD) of ligand between predicted and crystallized is 0.12 nm. Both the large energy gap (larger than 15 kJ/mol) between the largest cluster (ligand binds to the natural ligand binding site) and the others and the small RMSD value between predicted and crystallized show that the using docking methodology is satisfactory to the study. Then, 4,4’-DDE and CB-153 were docked to AR/LBD with 100 independent runs for each case. 6 and 5 clusters were obtained for 4,4’-DDE and CB-153, respectively.

**Figure 3:**
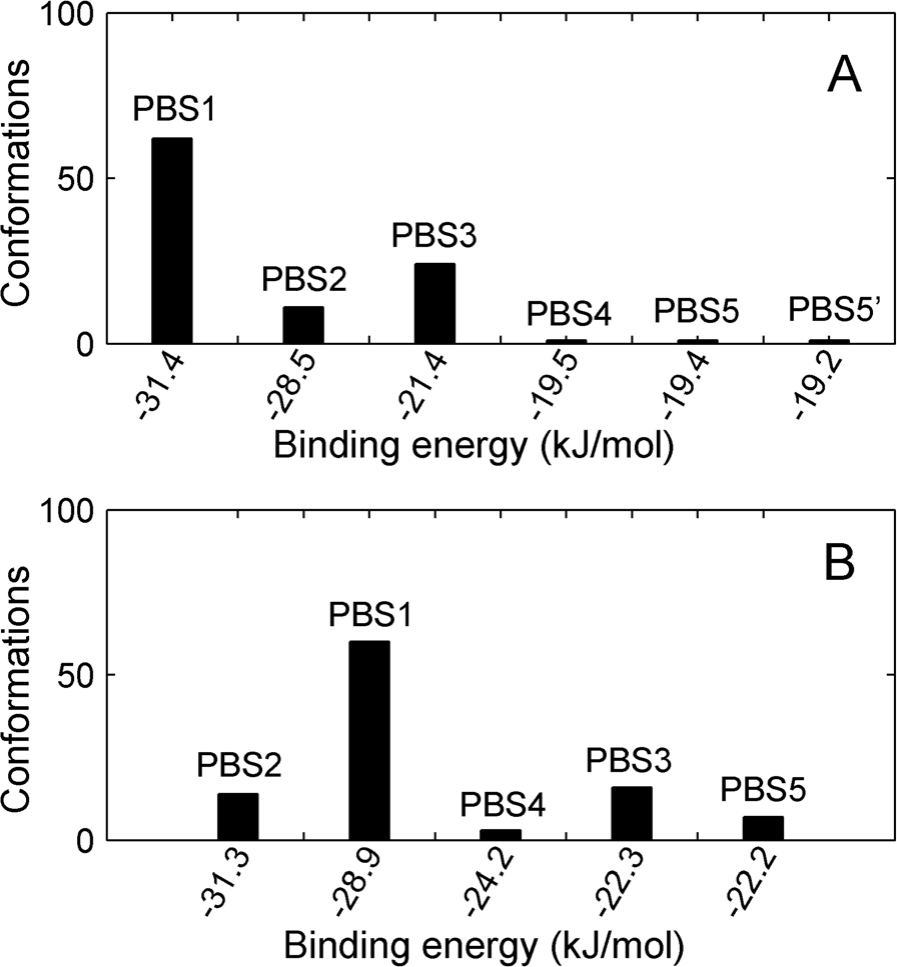
**Docking results of POPs with AR/LBD.** Generated 100 docking poses were clustered by root mean square (RMS) difference with a cutoff value 0.2 nm for each case. The binding energy shown in the x-axis is the mean value of each cluster. **(A)** is for 4,4’-DDE and **(B)** is for CB-153. The chosen models are marked by PBS1-5 according to the positions of binding sites.

**Figure 4:**
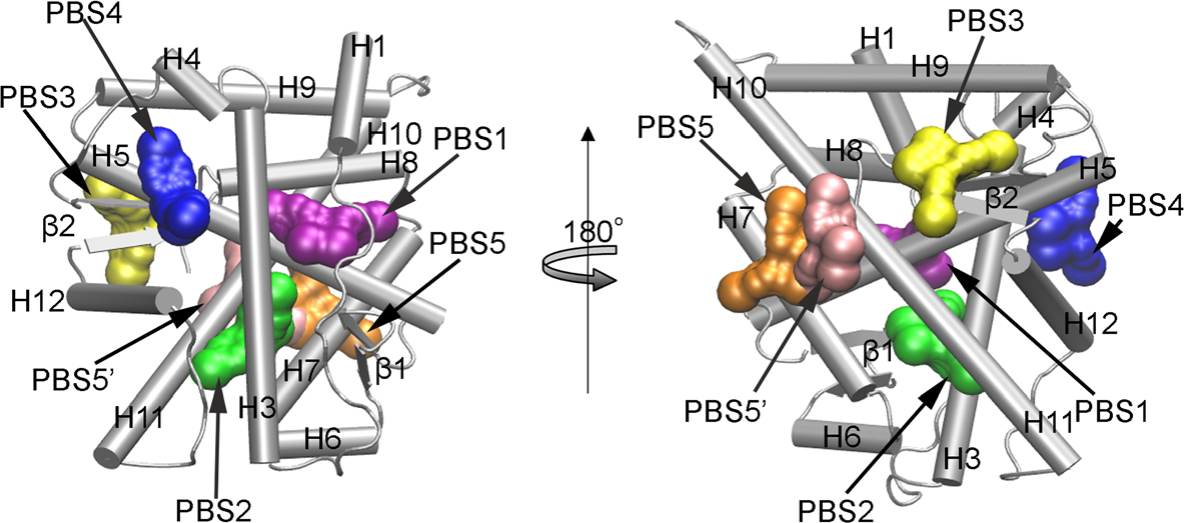
**Predicted potential binding sites for POPs.** Binding sites are determined by the docking study between AR/LBD and POPs. PBS1 is a cleft near the N-terminal of the receptor. PBS2 is also known as the bind site of the natural ligand, which is deep inside the receptor. PBS3 is a pocket on the surface near the C-terminal of the receptor. PBS4 is in AF2 region. PBS5 is on the surface of between H7 and H10. The figure on the right panel is obtained by rotating the left panel along z-axis with 180 degrees.

As shown in Figure 3A for 4,4’-DDE, the first three clusters comprise the majority of the total docking generations with a occupancy of 97%. The occupancies of the first three clusters are 62%, 11%, and 24% with average values of binding energy −31.4, -28.5, and −21.4 kJ/mol, respectively. The remaining three clusters consist of only one docking pose for each case. Potential binding sites (PBSs) corresponding to the six clusters are illustrated in Figure 4. PBS1 is a novel binding pocket with cleft formed by H1, H3, H5 and H8. PBS2, a hydrophobic pocket deep inside the receptor, is the same binding site of the androgens like DHT, which is also the binding site of antagonists. PBS3 is a pocket on the surface near the C-terminal comprised of β2 and H4. PBS4 is in the AF2 region, a hydrophobic surface for the binding of coactivators. PBS5 is on the surface of helix 7 and 10. The last potential binding site, PBS5’ , is quite near to the PBS5 with a similar value of binding free energy. We consider these two possible bind sites as the same one.

Figure 3B shows the cluster composition for CB-153 docking poses. Remarkably, the same five potential binding sites are also observed for CB-153. In Figure 3B, each cluster is marked according to the location of the potential binding site shown in Figure 4. Similar to the case of 4,4’-DDE, the cluster corresponding to PBS1 is the largest one with a occupancy of 60%. Differently, the PBS with the lowest binding free energy is PBS2, the natural ligand binding site. The occupancies of PBS4 and PBS5 are larger than those for 4,4’-DDE.

To obtain more favorable binding modes and also the inhibition mechanism of POPs with AR/LBD, docking pose with the lowest binding free energy in each cluster has been chosen for further MD study and MM/PBSA analysis.

### Conformational stability of Simulations

**Figure 5:**
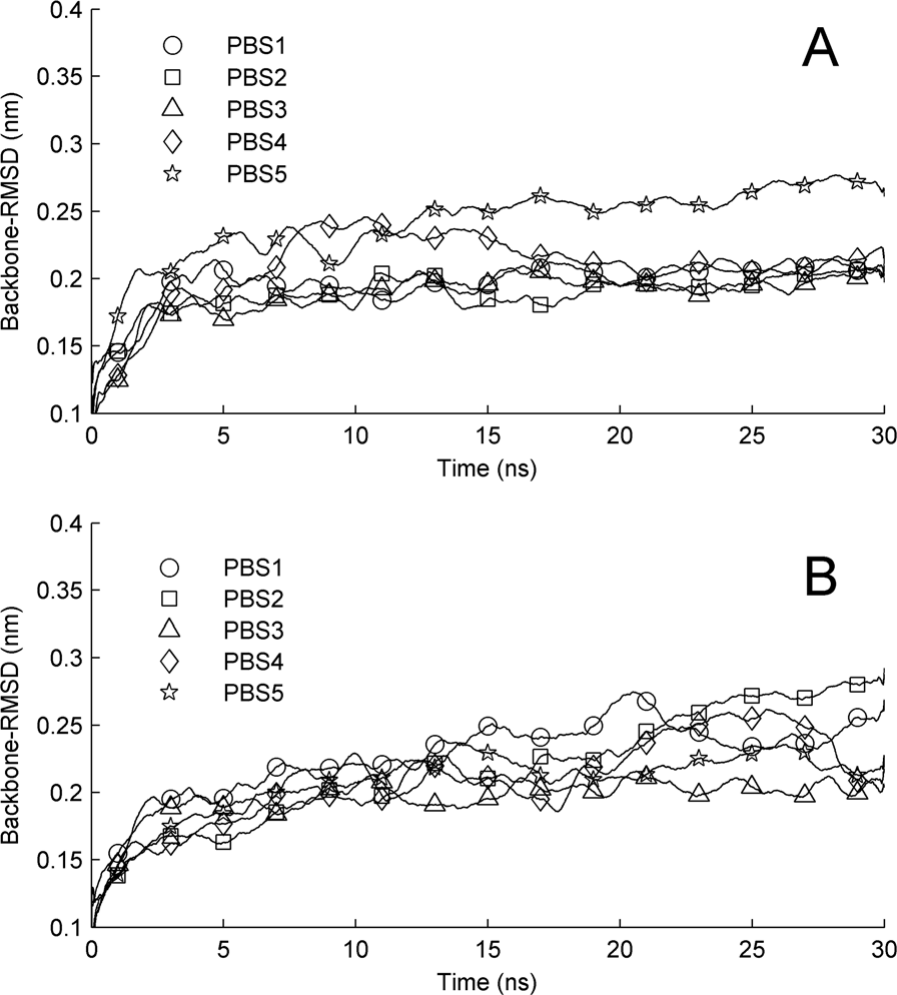
**Time evolutions of backbone RMSD of AR/LBD, based on 30 ns MD simulation for each AR/LBD-ligand complex. (A)** for the three binding modes of 4,4’-DDE with LBD and **(B)** for the case of CB-153.

Ten MD runs with 30 ns were carried out on selected AR/LBD-POPs complex structures. In order to investigate the stability of the receptor after ligand binding, we calculated temporal evolutions of backbone RMSD of the receptor. As shown in Figure 5A for predicted AR/LBD-4,4’-DDE complexes, backbone-RMSDs for the first three runs reach stable values after about 3 ns and stay around 0.2 nm in the remaining simulations. The value of backbone-RMSD of model 4 keeps rising to 0.25 nm in the first 10 ns and returns back to around 0.2 nm at the end of the simulation. For the model 5, the value of backbone-RMSD increases rapidly up to 0.21 nm after the simulation. When the equilibrium is reached, the values of backbone-RMSD are around 0.25 nm, which is larger than other models. Figure 5B shows the temporal evolutions of backbone-RMSDs of receptor in predicted AR/LBD-CB-153 complexes. Similar to that observed in Figure 5A, the values of backbone-RMSD of all runs continue rise in the first 3 ns. Then, the values of backbone-RMSD for PBS1, PBS2, PBS4, and PBS5 keep rising and fluctuate around 0.23 nm in the remaining simulations. The backbone-RMSD for PBS3 reaches stable with a smaller value around 0.2 nm.

**Figure 6:**
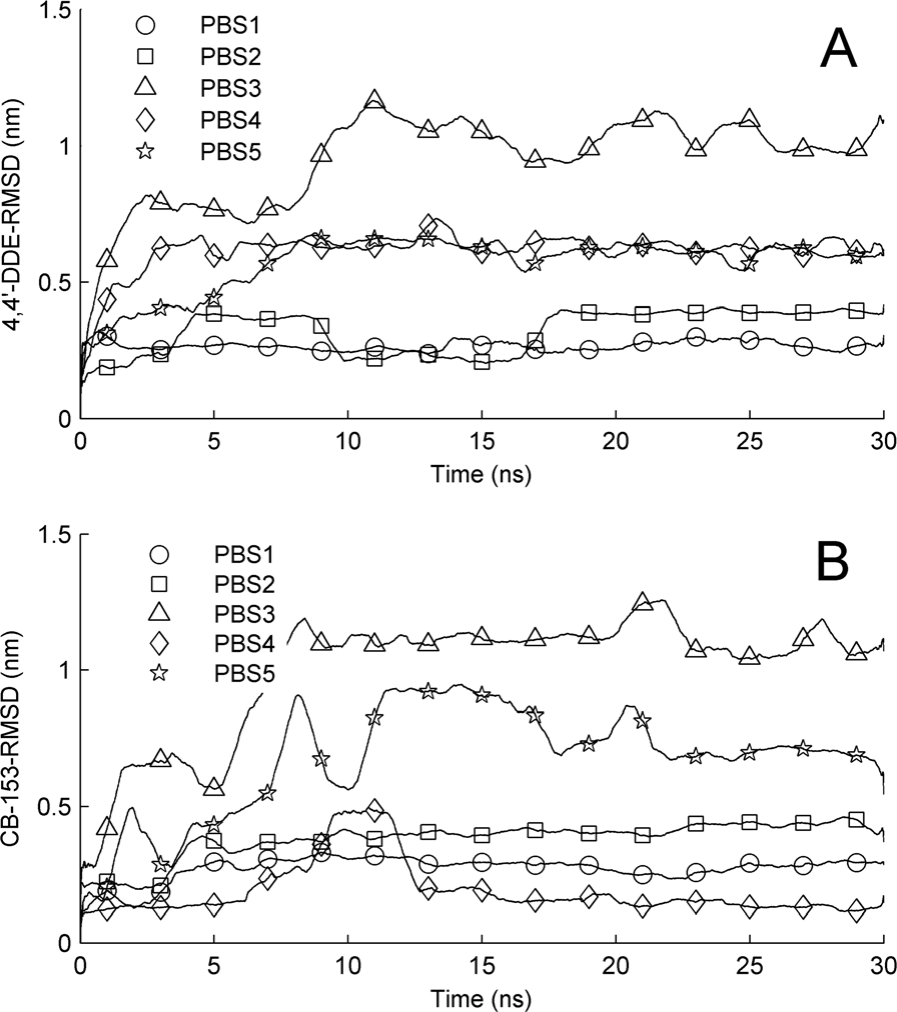
**Time evolutions of ligand RMSD, calculated by superimposing backbone atoms of the receptor AR/LBD. (A)** for AR/LBD-4,4’-DDE complexes and **(B)** for AR/LBD-CB-153 complexes. The corresponding time evolutions of receptor RMSD are shown in Figure 5.

To investigate the stability of ligands in binding sites, we calculated RMSDs of ligands as a function of time by superimposing backbone atoms of the receptor. As shown in Figure 6A for AR/LBD-DDE complexes, the RMSD of 4,4’-DDE in PBS1 rapidly increases up to 0.3 nm and then keep stable in the whole simulation. For the case of PBS2, the value of RMSD slowly rises to 0.4 nm in the first 5 ns and keeps stable for about 5 ns, followed by a decrease to 0.3 nm. At 16 ns, the RMSD of 4,4’-DDE rises again reaching up to 0.4 nm and keeps stable in the rest of the simulation. The whole RMSD evolution displays an indented curve which dues to the local rearrangement after ligand binding. Significantly, the RMSD of 4,4’-DDE in PBS3 rises quickly up to a relatively large value of 0.8 nm in the first 2 ns and keeps stable for about 5 ns. Then it rises again and fluctuates around a value of 1.0 nm in the remaining time. The ligand RMSDs for PBS4 and PBS5 rise up to a value of 0.7 nm and keep stable in whole simulations. The large values of RMSD observed in model 3, 4, and 5 indicate that new binding patterns in those binding sites have been achieved during MD simulations. The temporal evolutions of ligand RMSD in AR/LBD-CB-153 complexes are shown in Figure 6B. Similar to that observed for 4,4’-DDE, the values of ligand RMSD for PBS1 and PBS2 increase up to 0.2 nm and 0.3 nm, respectively, in the first 5 ns and then they keep stable in the following 25 ns simulations. Relative large values of RMSD have been observed after several nanosecond simulations for PBS3 and PBS5. Differently, an indented curve was observed for the evolution of ligand RMSD when CB-153 binds to PBS4. The equilibrium value of ligand RMSD for PBS4 is very small with a value around 0.1 nm. Overall, ligands binding in PBS1 and PBS2 are quite stable during MD simulations, while large motions were observed when ligands are located at PBS3, PBS4, and PBS5, which are on the surface of the receptor.

### Binding free energy

The binding free energies of predicted AR/LBD-POPs complexes were estimated by MM/PBSA approach, which has been widely used on protein-ligand complexes and compared with experimental measurements [45,50]. The values are reported in Table 1. For 4,4’-DDE, the binding mode of PBS1 is the most stable one with a binding free energy value of −198.9 kJ/mol, which is lower than that of the mode binding to natural ligand binding site PBS2 (−177.0 kJ/mol). On the contrary, for the CB-153 ligand, the binding free energy in PBS2 (−192.9 kJ/mol) is visibly lower than that at PBS1 (−142.8 kJ/mol). Besides, low values of binding free energy were also observed for the two ligands (−128.8 kJ/mol for 4,4’-DDE and −121.7 kJ/mol for CB-153) when they bind to PBS4. However, the binding free energy values of both ligands at PBS3 are significantly larger than those binding in previous binding sites. For the last potential binding site, PBS5, the value of binding free energy for 4,4’-DDE (−97.5 kJ/mol) is comparable to that of PBS3 and the value for CB-153 (−120.7 kJ/mol) is comparable to that of PBS4. According to the results of binding free energy, it comes out that 4,4’-DDE prefers to bind to PBS1 while CB-153 prefers to bind to PBS2. It is noted that PBS2 is the binding site for natural ligands, and a low binding free energy was observed when ligands bind to this binding site. Interestingly, we also found that the binding free energies of ligands binding in PBS1 are comparable to or even lower than those for PBS2, indicating a possible alternative binding site through which POPs display their disrupting effects. Besides, low values of binding free energy were also observed when POPs bind to PBS4 which corresponds to AF2 region, suggesting another possible inhibition mechanism.

**Table 1:** Binding free energies of 4,4’-DDE, CB-153, and DHT with AR/LBD

**Binding mode**	**Δ*E***_***elec***_	**Δ*E***_***vdW***_	**Δ*G***_***PB***_	**Δ*G***_***SA***_	***-T*Δ*S***	**Δ*G***_***b***_
**4,4’-DDE/PBS1**	−18.8(7.8)	−206.2(9.5)	22.9(3.18)	−10.9(0.5)	14.1	−198.9
**4,4’-DDE/PBS2**	−3.9(1.8)	−196.8(9.1)	9.4(1.9)	−11.2(0.5)	25.5	−177.0
**4,4’-DDE/PBS3**	−10.9(6.1)	−119.7(16.8)	11.8(6.7)	−6.4(0.9)	29.6	−95.6
**4,4’-DDE/PBS4**	−2.1(2.5)	−147.2(12.4)	8.4(2.2)	−8.3(0.7)	20.4	−128.8
**4,4’-DDE/PBS5**	−4.0(4.9)	−120.0(20.4)	8.8(2.7)	−6.2(1.4)	23.9	−97.5
**CB-153/PBS1**	−5.6(5.8)	−164.8(9.8)	25.7(4.2)	−9.1(0.6)	11.0	−142.8
**CB-153/PBS2**	−4.3(2.2)	−197.8(9.2)	10.2(1.9)	−11.3(0.5)	10.4	−192.9
**CB-153/PBS3**	−2.8(4.5)	−121.8(18.9)	13.3(2.5)	−6.6(1.0)	14.6	−103.3
**CB-153/PBS4**	0.1(0.8)	−131.2(8.8)	7.2(2.1)	−8.2(0.6)	10.4	−121.7
**CB-153/PBS5**	−4.3(3.8)	−130.3(15.0)	12.9(3.5)	−7.2(1.3)	8.2	−120.7
**DHT/PBS2**	−41.8(12.1)	−195.5(9.9)	12.6(11.5)	−10.5(0.5)	26.2	−209.0

The unit is in kJ/mol and values in parentheses are standard deviations of average.

An advantage of MM/PBSA approach is that it allows us to assess the contributions of each term in the free energy function (Eq. 1). The contributions of the gas-phase molecular mechanics energy and the solvation components are listed in Table 1. Since structures (monomers or complex) used for MM/PBSA calculations are derived from the same MD trajectories, the contributions of internal term of the molecular mechanics (ΔE_int_) are set to zero. Favorable contributions from electrostatic term (ΔE_elec_) have been observed for all binding modes, with values of −2.1 ~ −18.9 and −0.1 ~ −5.6 kJ/mol for 4,4’-DDE and CB-153, respectively. Significantly, values of van der Waals (ΔE_vdW_) energy are from −119.7 to −206.2 kJ/mol for 4,4’-DDE and from −121.8 to −197.8 for CB-153, indicating large favorable contributions to the final binding free energies (ΔG_b_). The results are consistent with the hydrophobic character of the ligands. At variance, unfavorable contributions were detected for polar solvation energy (ΔG_PB_). Values of ΔG_PB_ for both ligands binding in PBS1 are larger than those binding to the other binding sites; this might be due to rearrangements of some charged residues in PBS1 after the ligand binding (also see below). Low values of nonpolar solvation energy (ΔG_SA_), ranging from −9.1 to −11.3 kJ/mol, were observed for both ligands when they bind to PBS1 or PBS2. While higher values of ΔG_SA_ were detected when POPs bind to other possible bind sites, attributing to the smaller interface between ligands and receptor in these binding modes. The entropic results show that the configurational entropic component is unfavorable to the ligand binding for all binding modes.

Since natural ligand binding site (PBS2) has been considered as one of the potential binding sites of POPs, for comparison, the binding free energy of the receptor with the natural ligand DHT was also calculated based on the crystal structure of complex AR/LBD-DHT with PDB ID: 1I37 [17]. 10 ns MD simulation was performed with the same setting described in the Method Section. 500 snapshots taken from the last 5 ns stable trajectory were used for the MM/PBSA calculation. The results are also listed in Table 1. The binding free energy of DHT to AR/LBD is −209.0 kJ/mol which is slightly lower than those of 4,4’-DDE (−177.0 kJ/mol) and CB-153 (−192.9 kJ/mol). The detailed energetic components show that the lower binding free energy of DHT is mainly due to the electrostatic term (ΔE_elec_). This is in agreement with the evidence of several hydrogen bonds forming between DHT and AR/LBD in the crystal structure [17].

### Predicted binding modes

**Figure 7:**
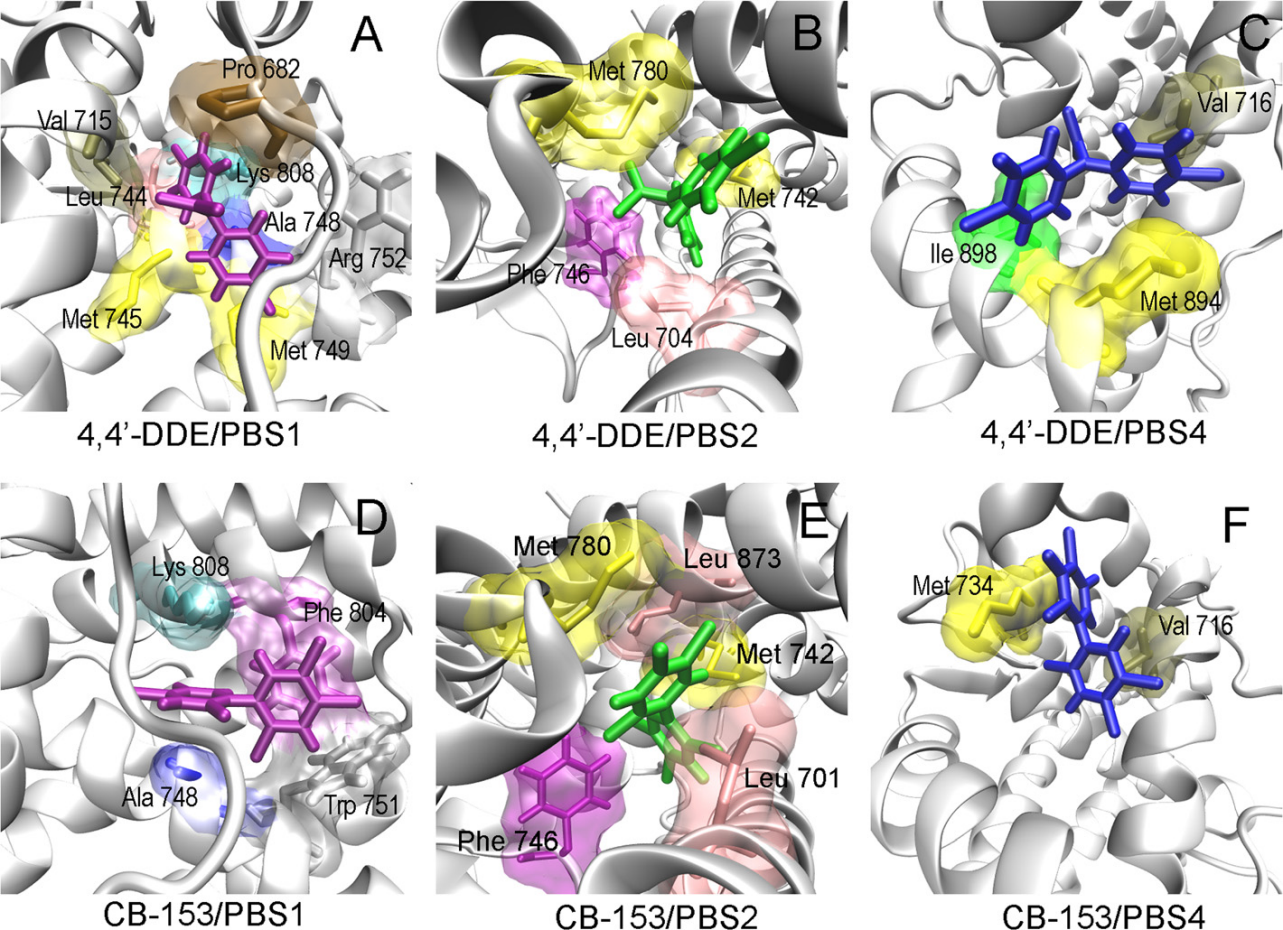
**Detailed interactions of predicted binding modes of POPs with AR/LBD, obtained by averaging over the last 100 ps of each MD run. (A)**, **(B)** and **(C)** correspond to 4,4’-DDE binding to AR/LBD on PBS1, PBS2 and PBS4, respectively. **(D)**, **(E)** and **(F)** correspond to CB-153 binding to AR/LBD on PBS1, PBS2 and PBS4, respectively. Contact residues (marked in the figure) are determined by the LIGPLOT program with a cutoff value 0.4 nm. Both contact residues and POPs are represented with the licorice model.

To investigate detailed interactions between POPs and the receptor, the structure of each binding mode was averaged over last 100 ps from MD simulation. In Figure 7, we illustrate the three most possible binding modes (PBS1, PBS2, and PBS4) for each ligand. The other two possible binding modes for each case are reported in Additional file 1: Figure S1. Contacting residues of the ligand were determined by LIGPLOT program with a cutoff value of 0.4 nm [51].

It is found that POPs can bind to PBS1 with high affinities, especially for 4,4’-DDE. As shown in Figure 7, the binding pocket of 4,4’-DDE/PBS1 is mainly formed by the hydrophobic residues including Pro682, Val715, Leu744, Met745, Val748, and Met749. Additionally, two positive charged residues, Arg752 and Lys808, also interact with the ligand. Interestingly, Lys808 forms a cation-π interaction with a benzyl group of DDE, which might be important for the binding of 4,4’-DDE. For the case of CB-153/PBS1, the ligand is inserted into the cleft with ligand-contacting residues Ala748, Trp751, Phe804, and Lys808. A cation-π interaction between Lys808 and a benzyl group of CB-153 was also observed. Besides, a π-π packing is formed between Phe804 and the other benzyl group of CB-153 (also see Figure 7D). As shown in Figure 3, PBS1 is far away from AF2 region. The ligands binding to this site might disrupt the receptor’s function through some allosteric effects, which needs further experimental confirmation.

In PBS2, both 4,4’-DDE and CB-153 are buried inside the hydrophobic cavity formed by H3, H5, H11, H12, and β1. The binding pocket of 4,4’-DDE/PBS2 consists of four hydrophobic residues, Leu704, Met742, Phe764, and Met780. Interestingly, almost the same hydrophobic pocket was found for CB-153/PBS2 which is formed by resides, Leu704, Met742, Phe764, Met780, and Leu873. Besides that, the orientations of the two ligands in the binding pocket are similar with one benzyl group inserting between Phe764 and Met742 and the other one toward the Met780. Since DHT acts as an agonist via binding to PBS2, the comparison of binding modes between POPs and DHT could provide clues to understanding of the inhibitory mechanism of 4,4’-DDE and CB-153 against AR. The detailed interactions between DHT and AR/LBD and the comparison with POPs can be found in supplementary material (Additional file 1: Figure S2), revealing different binding patterns of DHT and POPs corresponding to their opposite behaviors agonist AR.

Different to the above two binding sites, PBS4 is a hydrophobic binding pocket formed by H3, H5, and H12 on the surface of the receptor (also see Figure 2 and Figure 4). Detailed contacts of ligands with the receptor are also displayed in Figure 7. 4,4’-DDE binds to PBS4 by anchoring one benzyl group between Val716 and Met894 and the other between Met894 and Ile898. For CB-153 as shown in Figure 7F, interactions are mainly focused on Val716 on H3 and Met734 on H5. As illustrated in the section of Introduction, AF2 region has been identified to be a binding site of some antagonists for AR as well as some other members of NR. Since PBS4 is in the AF2 region corresponding to the interface of AR with coactivators, binding of POPs to this binding site would interrupt directly interactions between AR and its coactivators.

## Conclusions

In the present study, molecular docking and MD simulation were performed to probe the binding modes of two most widespread POPs, 4,4’-DDE and CB-153, with AR/LBD. Several potential binding sites including natural ligand binding site (PBS2) and AF2 (PBS4) have been detected and analyzed. MD simulations of the docking poses have allowed us to show that POPs form stable complexes with AR/LBD. The binding free energies of POPs and an agonist DHT with AR/LBD were estimated using MM/PBSA approach. The results reveal that the binding free energies of POPs binding to PBS2 are comparable with those of AR/LBD-DHT complex. Significantly, a novel potential binding site PBS1 possesses similar binding free energies to those of PBS2 with POPs binding. Our study illustrated the endocrine disrupting mechanism of POPs, which would also be useful for designing new drugs with AR as a target.

## Abbreviations

POPs: Persistent organic pollutants; EDCs: Endocrine disrupting chemicals; 4,4’-DDE: Dichlorodiphenyldichloroethylene; PCBs: Polychlorinated biphenyls; CB-153: 2,2’,4,4’,5,5’-Hexachlorobiphenyl; DHT: 5α-Dihydrotestosterone; NR: Nuclear receptor; AR: Androgen receptor; LBD: Ligand binding domain; AF2: Activation function 2; PBS: Potential binding site; MD: Molecular dynamics; MM/PBSA: Molecular mechanics/Poisson-Boltzmann surface area.

## Competing interests

The authors declare that they have no competing interests.

## Authors’ contributions

XJX contributed to the design of the study, carried out the simulations, analyzed results and wrote the manuscript. JGS, ARB, and CXW contributed to the design of the study and critically revised the manuscript. SC, ML, YZ, and WZC participated in the design and helped to draft the manuscript. All authors have read and approved the final manuscript.

## Supplementary Material

Additional file 1: Figure S1 Detailed interactions of predicted binding modes of POPs with AR/LBD at PBS3 and PBS5, obtained by averaging over the last 100 ps of each MD run. **Figure S2.** Binding mode of DHT with AR/LBD and the comparison with POPs. Click here for file
