# Supplementary material for: Detection of persistent organic pollutants binding modes with androgen receptor ligand binding domain by docking and molecular dynamics
Source: BMC Struct Biol. 2013 Sep 22;13:16. doi: 10.1186/1472-6807-13-16 (PMC3848780; doi:10.1186/1472-6807-13-16)
Supplement: Additional file 1: Figure S1 — Detailed interactions of predicted binding modes of POPs with AR/LBD at PBS3 and PBS5, obtained by averaging over the last 100 ps of each MD run. Figure S2. Binding mode of DHT with AR/LBD and the comparison with POPs. [file 1472-6807-13-16-S1.pdf]

Supplementary Information

Detection of persistent organic pollutants binding modes with androgen receptor ligand binding domain by docking and molecular dynamics

Xian Jin Xu[1], Ji Guo Su[2], Anna Rita Bizzarri[3], Salvatore Cannistraro[3], Ming Liu[4], Yi Zeng[1], Wei Zu Chen[1], Cun Xin Wang[1§]

[1]College of Life Science and Bioengineering, Beijing University of Technology, Beijing 100124, China

[2]College of Science, Yanshan University, Qinhuangdao 066004, China

[3]Biophysics and Nanoscience Centre, Facoltà di Scienze, Università della Tuscia, Largo dell’Università, 01100 Viterbo, Italy

[4]Beijing Institute of Biotechnology, Beijing 100071, China

[§]Corresponding author: Cun Xin Wang. E-mail: cxwangbjut@gmail.com Tel.: (+86)10-67392724, Fax: (+86)10-67392837

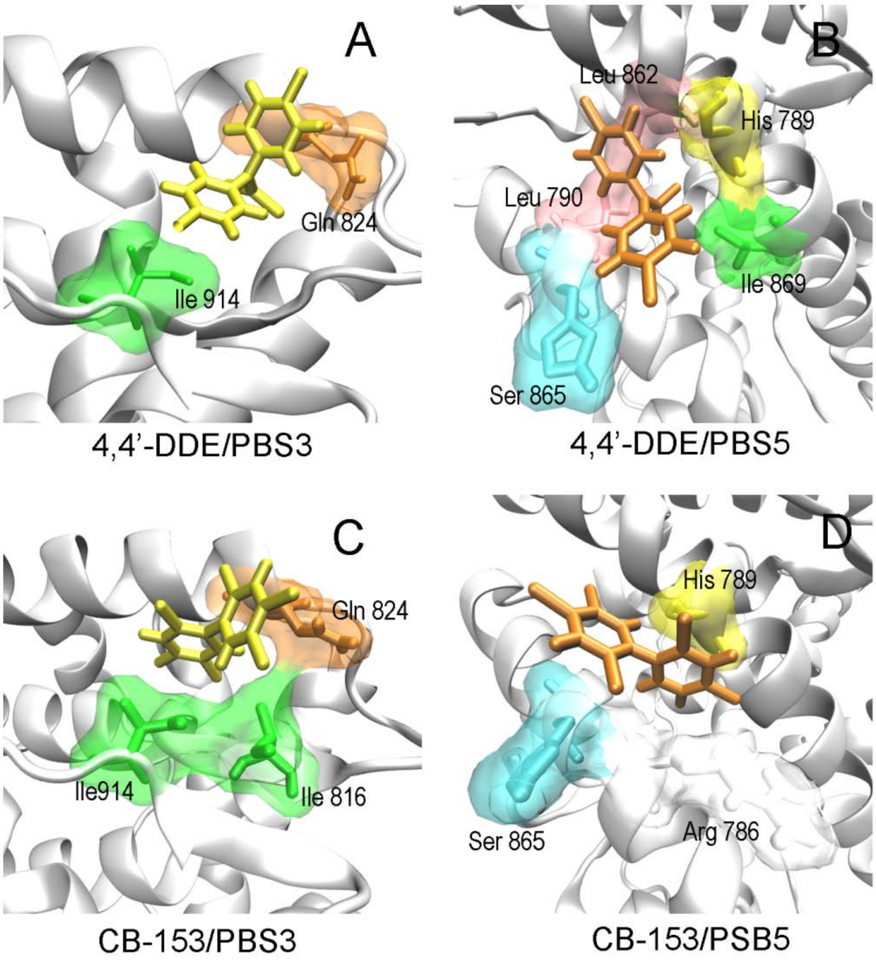

Fig. S1 Detailed interactions of predicted binding modes of POPs with AR/LBD at PBS3 and PBS5, obtained by averaging over the last 100 ps of each MD run. The contact residues (marked in the figure) are determined by the LIGPLOT program with a cutoff value 0.4 nm. Both contact residues and POPs are represented with the licorice model.

Predicted binding modes 3 and 5

PBS3 is a binding pocket, formed by β2 and H4, locating at the surface near the C-terminal of the LBD. The detailed contacts with the ligand are displayed in Fig. S1. 4,4'-DDE binds to PBS3 through inserting a dichloro group into a cleft between Gln824 and Ile914. The two benzyl groups of 4,4'-DDE are attached to the two residues, respectively. For the CB-153/PBS3, besides the Gln824 and Ile914, another contact residue Ile816 was detected. Different to the binding pattern of 4,4'-DDE/PBS3, CB-153 binds to PBS3 through anchoring a benzyl group into the pocket formed by the ligand-contacting residues (see Fig. 6 for more details).

PBS5 is on the surface of H7 and H10. As shown in Fig. S1B, 4,4'-DDE binds to PBS3

through inserting a dichloro group into a pocket formed by residues His789, Leu790, Leu862, Ser865, and Ile869. The binding pocket for CB-153 is formed by residues Arg786, His789, and Ser865. It is worth to note that corresponding regions of H7 and H10 in several other steroid hormone receptors are on the interface of homodimers formation [1]. The binding of POPs to PBS5 would disrupt the dimerization progress of AR.

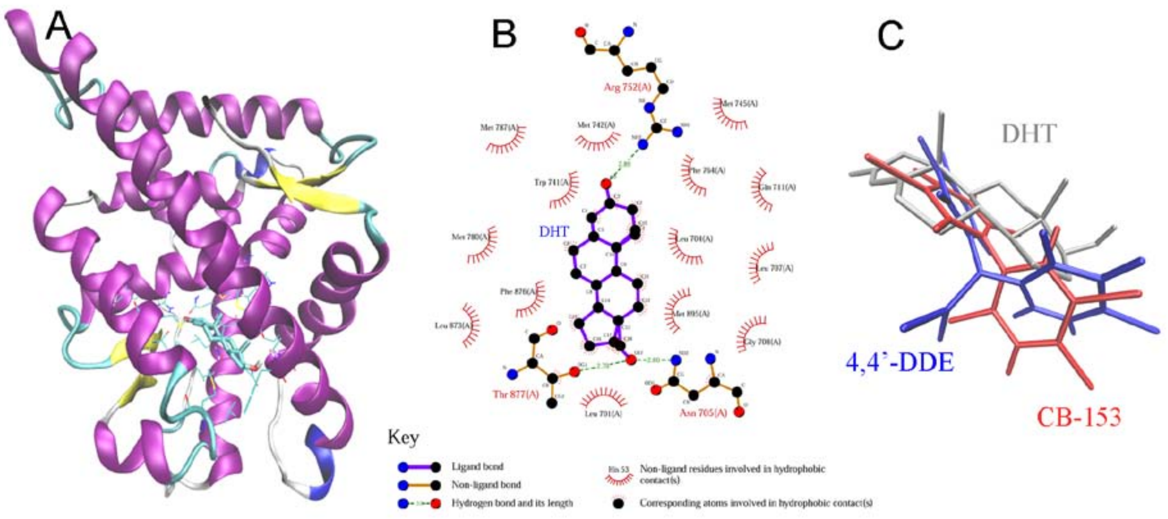

Fig. S2 Binding mode of DHT with AR/LBD and the comparison with POPs. (A) The complex structure of DHT-AR/LBD was created based on pdb entry 1i37. DHT is represented with the licorice model. Contact residues shown with line model in (A) are determined by the LIGPLOT program (B). Three hydrogen bonds are observed between DHT and three contact residues Asn705, Arg752, and Thr877. (C) Binding modes of POPs in PBS2 are compared to DHT by superimposing backbone atoms of AR/LBD receptor.

Comparison of binding modes of DHT and POPs in PBS2

The detailed interactions and contact residues of AR/LBD with DHT are illustrated in Fig. S2 A and B. The binding site involves a large amount of hydrophobic residues, such as Leu701, Leu704, Leu707, Gly708, Trp741, Met742, Met745, Phe764, Met780, Met787, Met873, Phe876, and Met895. Significantly, DHT forms three hydrogen bonds with receptor via three residues, Asn705, Arg752, and Thr877. Comparing the contact residues of DHT with those of 4,4'-DDE and CB-153 when they bind in PBS2, it is found that contacts residues of the latter are mostly involved in the former one. However, 4,4'-DDE and CB-153 cannot form hydrogen bonds with receptor. To further compare the orientation of ligands in binding site, backbone atoms of three

complexes are superimposed and ligands are shown in Fig. S2 C. 4,4'-DDE and CB-153 share a similar orientation and location, while their orientations are obviously different to DHT with one benzyl group been almost perpendicular to the surface of DHT molecule. The different contact residues and orientations are supposed to be responsible for the opposite behaviors of DHT and POPs agonist AR.

1. Huang P, Chandra V, Rastinejad F: **Structural overview of the nuclear receptor superfamily: insights into physiology and therapeutics**. *Annu rev physiol* 2010, **72**:247-272.
